# Ecological indicator values reveal missing predictors of species distributions

**DOI:** 10.1038/s41598-019-39133-1

**Published:** 2019-02-28

**Authors:** Daniel Scherrer, Antoine Guisan

**Affiliations:** 1 Department of Ecology and Evolution, University of Lausanne, Biophore, CH-1015 Lausanne, Switzerland; 2 Institute of Earth Surface Dynamics, University of Lausanne, Géopolis, CH-1015 Lausanne, Switzerland

## Abstract

The questions of how much abiotic environment contributes to explain species distributions, and which abiotic factors are the most influential, are key when projecting species realized niches in space and time. Here, we show that answers to these questions can be obtained by using species’ ecological indicator values (EIVs). By calculating community averages of plant EIVs (397 plant species and 3988 vegetation plots), we found that substituting mapped environmental predictors with site EIVs led to a doubling of explained variation (22.5% to 44%). EIVs representing light and soil showed the highest model improvement, while EIVs representing temperature did not explain additional variance, suggesting that current temperature maps are already fairly accurate. Therefore, although temperature is frequently reported as having a dominant effect on species distributions over other factors, our results suggest that this might primarily result from limitations in our capacity to map other key environmental factors, such as light and soil properties, over large areas.

## Introduction

Species distribution models^1–3^ (SDMs, also termed habitat suitability models or ecological niche models) establish a statistical relationship between environmental variables (predictors) and observed species occurrences to estimate the realized environmental niche (sensu Hutchinson) and predict the realized or potential distribution of species^4–6^. Over the last two decades, the popularity of these models grew spectacularly^7^ while technical developments allowed predictions at increasingly fine spatial and temporal resolutions^8–13^. However, despite accumulated knowledge on the factors influencing niches and distributions, most SDMs still lack inclusion of several ecophysiologically important factors^9,13,14^. One reason for this limitation is that, although we know which variables are theoretically important (e.g. various expressions of heat, light, water and nutrients for plants), we still don’t know well what potential importance each variable has in explaining species distributions (e.g. climate versus substrate predictors for plants)^3^, and how well the commonly used environmental predictors truly reflect the species requirements^14^. Precise answers to such questions are still lacking, largely because we still have a very limited theoretical or experimental knowledge of species’ fundamental niches and how it relates to realized niches in the wild^8,15,16^, but also because we have limited tools to assess the relative importance of all potential environmental factors on the realized and potential distribution of species at various scales.

In contrast to SDMs that use environmental variables directly to predict species or community distribution, species’ ecological indicator values^17–19^ (EIVs) represent semi-quantitative estimates of environmental conditions suited for individual species based on comprehensive and usually long-lasting compilations of expert knowledge and field or experimental measurements. EIVs allow one to estimate environmental conditions in a given biotope based on the occurrence and abundance of its constituent plant species, by averaging their individual indicator values^20–24^. One must therefore clearly distinguish EIVs defined for a *species*, representing central estimates of univariate niche dimensions, from those obtained for a *site* based on the aggregated values of the constituent species in the target community. These site ecological indicator values (site EIVs) thus represent integrated signals of species-environment relationships at the level of communities, and as such provide robust information on the long-term environmental conditions characterizing the site^25,26^. As a result, these are commonly used in paleoclimatology to reconstruct past climate^27^, in studies focusing on shorter time periods of (anthropogenic) climate warming^28,29^ and in habitat monitoring studies for conservation purposes^30^. Yet, few studies so far have attempted - or even discussed - the possibility of using EIVs to assess the relative importance of environmental factors to predict species distributions, i.e. to combine SDMs and EIVs^23,24,31,32^.

**Figure 1:**
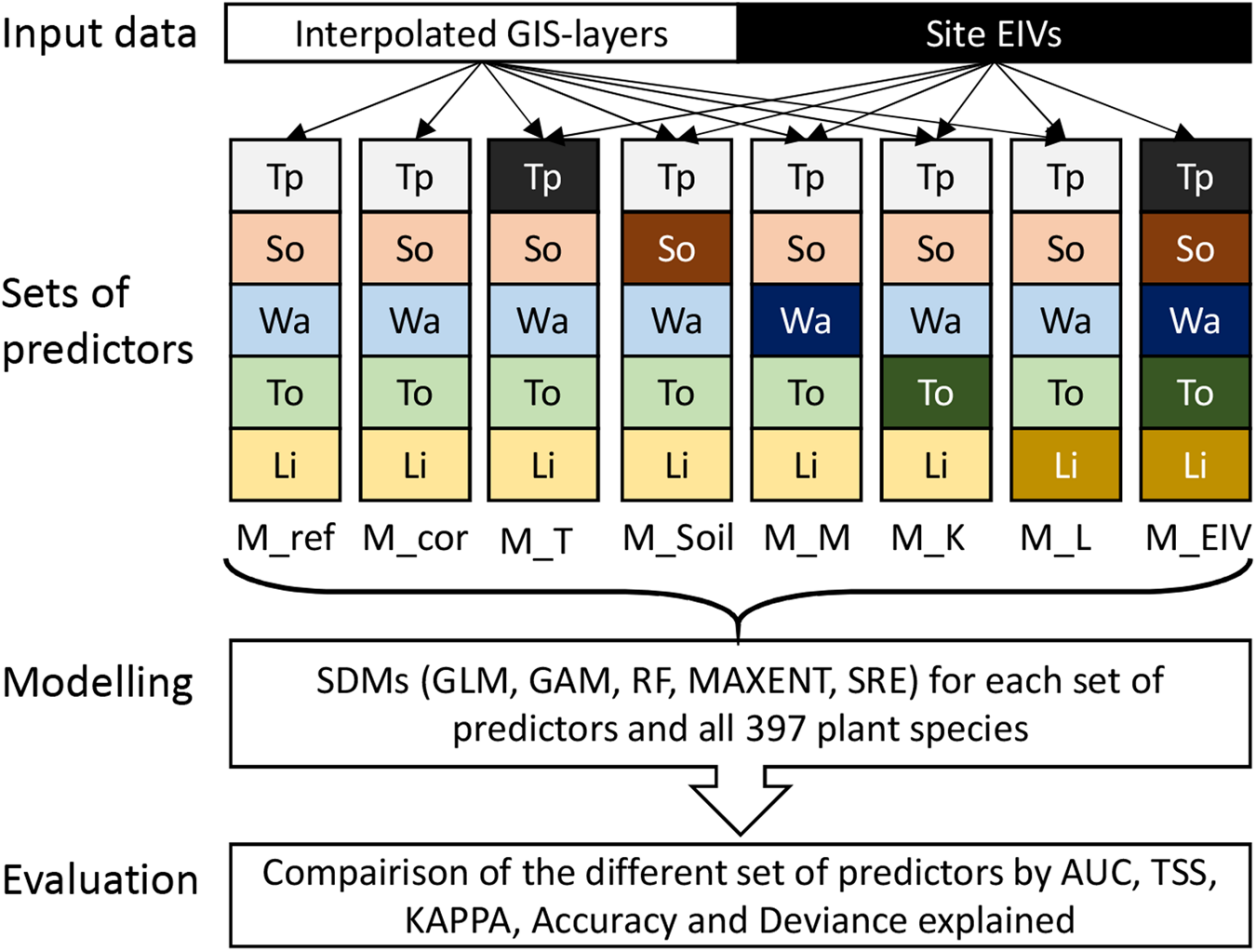
Framework used to test the predictive power of site EIVs. Light colored boxes represent environmental layers and dark colored boxes represent site EIVs. Tp = Temperature, So = Soil, Wa = Water, To = Topography and Li = Light. For details on predictors used see Table 1.

Here, we develop the idea that EIVs calculated at observation sites can shed light on which variables could be the most important drivers of species distributions^24^, and which ones should be further included in SDMs to capture more of the species’ environmental determinism. We show, using plants as a case study, how a modelling framework incorporating site EIVs (Fig. 1) can be used to evaluate the predictive power and suitability of those environmental variables most commonly used in SDMs (i.e. mainly climatic or topo-climatic predictors^14^). We hypothesize that plant EIVs represent more proximal^33^ descriptors of plant species distributions, allowing to estimate how much additional environmental variance can be potentially explained by SDMs, and ultimately approximate the maximum level of environmental determinism that can be quantified in SDMs at a given spatial resolution. We test and illustrate this idea using a dataset of 397 plant species from forests (3076 sites, r = 10 m) and open grasslands (912 sites, 8 × 8 m) occupying a complex landscape across an entire mountain region (700 km^2^), spanning wide environmental gradients (375 to 3210 m) at high spatial resolution (25 m).

## Results and Discussion

**Figure 2:**
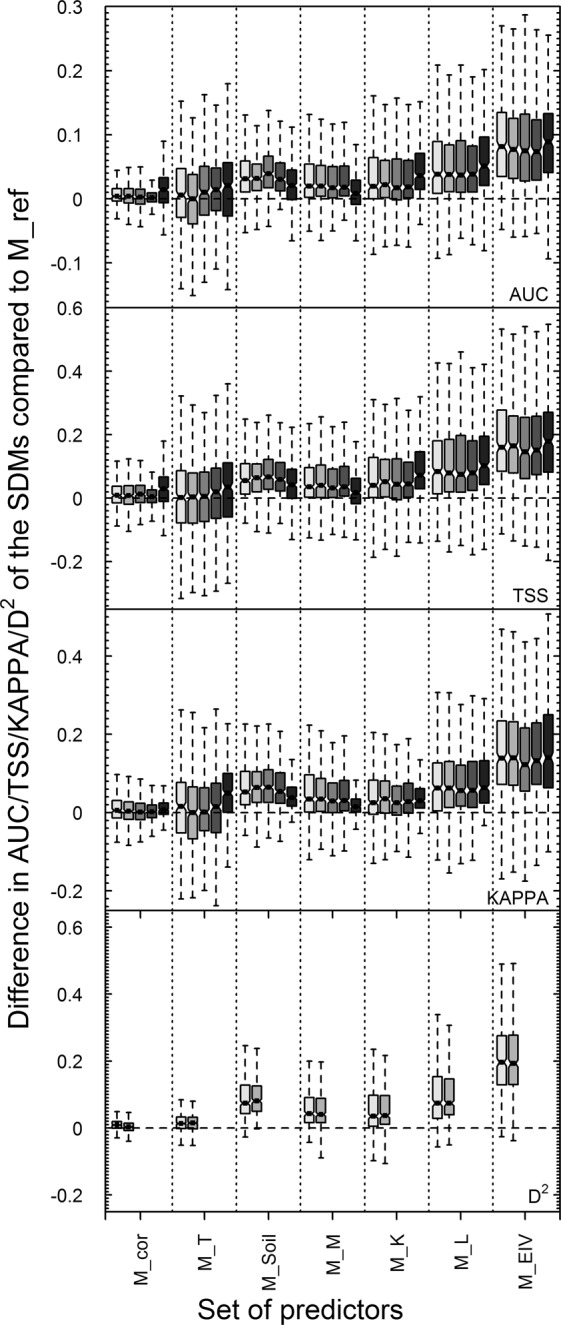
Differences in AUC, TSS, KAPPA and D2 of the seven different set of predictors compared to our reference model (M_ref, see Table 1 for details). The data shown is for 397 species evaluated on an independent ‘external’ data set of 1193 vegetation plots. The different shades of grey from light to dark represent GLM, GAM, RF, MAXENT and SRE models. The boxes represent the median and the 25/75 percentile and the whiskers are 2 SD.

Substitution of each individual environmental predictor by its corresponding site EIV improved average model performance for all metrics (Wilcox.test, p < 0.001; Fig. 2) compared to the reference model (M_ref; see Tables 1, S1 and S2) and for all variables except annual mean temperature. The strongest single variable effect was observed when the annual sum of solar radiation was replaced by the site EIV for light (M_L; +9–12%) and the model including all site EIVs always performed best (M_EIV; +17–22%, Fig. 2). This increase in average model performance was not the result of a few species highly benefiting from the addition of site EIVs, but was driven by a general improvement of individual SDMs for the large majority of species (Fig. 2). For instance, adding the site EIVs for soil (N, R or N + R), light (L) and moisture (M) improved the predictions for up to 93%, 86% and 81% of the species respectively (Fig. 2, Fig. S1). Furthermore, these improvements could not be caused by the addition of more variables (except for M_Soil; but see Fig. S1), as all models included the same number of predictors (or less in case of M_K), providing strong evidence that EIVs contain important additional information not accounted for by traditional environmental predictors.

**Table 1:** Different SDMs and their corresponding set of predictors.

Model	Temperature	Soil	Water	Topography	Light
M_ref	Annual mean temperature	pH	Annual precipitation	Topographic position & slope	Annual solar radiation
M_cor	Elevation	pH	Winter evapo-transpiration	Continuality index & slope	Cloud cover Jan
M_T	**T**	pH	Annual precipitation	Topographic position & slope	Annual solar radiation
M_Soil	Annual mean temperature	**N** & **R**	Annual precipitation	Topographic position & slope	Annual solar radiation
M_R	Annual mean temperature	**R**	Annual precipitation	Topographic position & slope	Annual solar radiation
M_N	Annual mean temperature	**N**	Annual precipitation	Topographic position & slope	Annual solar radiation
M_M	Annual mean temperature	pH	**M**	Topographic position & slope	Annual solar radiation
M_K	Annual mean temperature	pH	Annual precipitation	**K**	Annual solar radiation
M_L	Annual mean temperature	pH	Annual precipitation	Topographic position & slope	**L**
M_EIV	**T**	**N** & **R**	**F**	**K**	**L**

M_ref contains bio-climatic variables only, while M_EIV has site EIVs only. M_cor is based on the best correlation of bio-climatic predictors and site EIVs. All the other models are intermediate models where a single class of bio-climatic variables was replaced by the “corresponding” ecological indicator values. Letters in bold indicate site EIVs.

**Figure 3:**
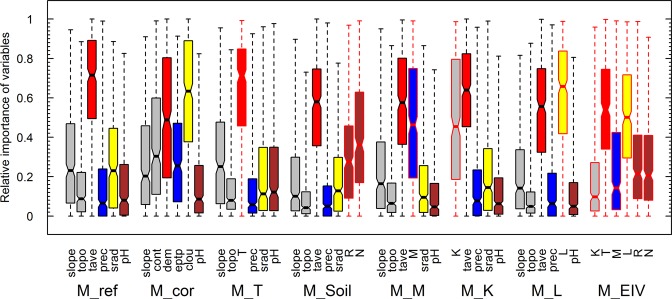
Relative importance of variables of eight sets of predictors (see Table 1 for details). The different colors represent different categories of predictors: grey for topographic, red for temperature, blue for water, yellow for light and brown for soil. The bars outlined in black represent environmental predictors and the bars outlined in red represent site EIVs. The boxes represent the median and the 25/75 percentile and the whiskers are 2 SD.

Results also showed that, apart from temperature, correlations were usually low between commonly used environmental variables and site EIVs (Table 2). Some less commonly used environmental variables, such as cloud cover or site water balance (precipitation minus potential evapotranspiration), were more strongly correlated with site EIVs. Yet, selecting these as predictors (M_cor) did not improve overall model performance (Fig. 2), but instead shifted variables’ importance (Fig. 3). The success of modelling the distribution of site EIVs in geographic space as a function of easily available environmental predictors (Table S3) strongly varied among the site EIVs, with very good predictions for site EIVs for temperature (T; cor = 0.94–0.96) and very poor predictions for site EIVs for continentality (K; cor = 0.41–0.53; Table S3). This strongly suggests that site EIVs represent more than simple combinations of easily available environmental predictors typically used in SDMs, and highlights their distinct and informative content. While site EIVs represent an integrated signal of the long-term local environmental conditions^17,19^ (depending on the ecosystem, from years to centuries), the commonly used environmental GIS layers represent interpolated or modelled trends not accounting sufficiently for local modifications of environmental conditions^32,34,35^, which seem to be very important when modelling species distributions at such fine spatial resolutions^36,37^.

**Table 2:** Spearman correlation of site EIV and commonly used environmental predictors.

Indicator value	Most commonly used	Best correlated
T (Temperature)	0.92 (annual temperature) 0.90 (degree days)	0.92 (elevation)
M (Moisture)	0.20 (precipitation) 0.06 (topographic wetness index)	0.39 (site water balance) 0.45 (winter-evapotranspiration)
L (Light)	0.13 (solar radiation) 0.10 (aspect)	0.49 (cloud cover)
R (pH)	0.42 (pH)	—
K (Continentality)	0.06 (slope) 0.07 (topographic position)	0.12 (continentality index)

For a detailed description of all 17 environmental variables see Table S4.

Apart from improving the average model performance, the replacement of environmental variables by site EIVs also significantly shifted the variable importance in all the models except those with the site EIV for temperature (M_T; Fig. 3). While temperature was by far the dominating variable in the reference model with topo-climatic predictors (M_ref) as well as in most SDM studies^14^, the inclusion of site EIVs led to a more even distribution of variable importance values (Fig. 3). The dominating importance of temperature might therefore be, at least partly, an artefact of the quality of the environmental variables used. The fact that the site EIV for temperature (T) (i) was highly correlated with annual mean temperature, (ii) was the only site EIV that could be modelled with good accuracy across the landscape using common environmental variables, and (iii) did not improve the predictive power of the models, suggests that the available high-resolution spatial information on temperature was already accurate, and little additional improvement was possible compared to the other environmental variables. In contrast adding the site EIVs for nutrients, soil pH, available water or light led to a general increase in model performance across all species (improved AUC for 93% of species). As getting information on soil and nutrients is time and money consuming^38^, most studies tend to use more easily available proxies, such as bedrock or soil types or coarse characteristics^39–41^. These maps are often based on coarser resolution vector maps and are therefore not able to reflect local variation in nutrient availability, soil depth or water holding capacity that would be needed to model at fine resolution (such as 25 m here). At fine resolution, the effects of soil structure and composition, and the resulting pH, water and nutrient contents, seem key to understand local community assembly^9,31,37,40,42,43^. Available water for plants is often expected to be reflected by seasonal precipitations in SDMs, which might not be very informative in landscapes with rugged topography, as the interpolated precipitation usually only show rough variation along altitude or latitude gradients^9,44^. Yet, soil moisture and water available for plants is known to be highly variable at locations with similar precipitation values but different topographic configurations and soil types^20,37^. These local differences in soil moisture were reflected in the corresponding site EIV (M), but did not seem to be captured by the interpolated precipitation maps, even in combination with topographic predictors. More complex modelling of soil moisture based on soil and topography would be needed to predict these variations^20,45^. Most site EIVs led to a similar increase in model performance across species and habitat types. Yet, the site EIV for light (L) had a much stronger effect in forested areas than in open grasslands (Appendix 1), highlighting the importance of information about available light in highly structured vegetation types with several layers of foliage such as forests or shrub lands^24^, with studies showing a strong improvement of model performance by adding canopy cover as a proxy for shading^24^.

The deviance explained by the SDMs using site EIVs as predictors (M_EIV; 44%) was almost doubled compared to our reference model (M_ref; 22.5%). However, even with the use of site EIVs capturing the local conditions at a high spatial resolution, a considerable proportion of unexplained variation remained in the models (D^2^ ranging from 5 to 80%). Assuming that our site EIVs are near-optimal environmental predictors for plants, this would lead to the conclusion that environment alone will never be sufficient to fully explain the distribution of all plant species at high spatial resolutions. Our results thus suggest that, at least for some species, large proportions of environmental variance can remain uncaptured by correlative models, and that other factors need to be accounted for, such as dispersal, population dynamics, biotic interactions and other community assembly processes^46,47^. Furthermore, there will likely always remain some stochasticity (e.g. in local extinction and colonization events) that cannot be accounted for by environmental conditions or by other deterministic factors. Additionally, here we only presented data on single species models, but it can be assumed that when modelling whole plant communities the unexplained deviance and stochasticity cumulate and become even higher^48,49^.

## Conclusion

Understanding and being able to predict species-environment relationships at different spatial and temporal scales is key to our biological understanding of the past, present and future functioning of ecosystems^48^. Here, we presented a new way of considering ecological indicator values (EIVs) in species distribution models (SDMs) as reasonable proxies of an optimal set of environmental predictor variables, allowing to unveil the level of environmental determinism that can theoretically be expected, based on expert knowledge, in correlative niche-based SDMs for plant species across a whole mountain region spanning wide environmental gradients at high resolution.

Our study revealed four key findings. (1) Significantly more environmental variance (up to 50% increase) could be explained in models based on site EIVs for the large majority of species, shedding light on which are the most effective environmental predictors and stressing the importance to develop several new environmental maps or improve existing ones, especially for soil nutrients and soil moisture but also for climatic parameters other than temperature, across whole regions. (2) Site EIVs for light and soil provided the greatest improvements, while site EIVs for temperature provided the smallest improvement. (3) Temperature was already largely accounted for in current models, and few additional improvements can be expected for this variable^12^. And finally, (4) even with all important EIVs incorporated, models kept variable parts of unexplained variance, depending on the species, which could correspond to a realistic estimate of the maximum predictable environmental determinism one might achieve in such models.

These findings pave the way toward using the most recent remote sensing technologies and advanced field measurements for developing spatially-explicit environmental predictors^14,50,51^, ultimately enabling plant SDMs to make a critical step forward. The best spatial resolution to use for this, however, still needs further investigations, as it likely depends on the modelled species. Another important research perspective is to evaluate whether the trend toward obtaining increasingly explanatory and predictive models at increased spatial resolution might come to a halt if more residual stochasticity is found as one moves toward very high spatial resolutions^12,52^.

## Material and Methods

### Case study area and vegetation data

We tested this new SDM approach based on EIVs in the Alps of the Vaud canton in Switzerland (46°10′ to 46°30′N; 6°60′ to 7°10′E), covering an area of ca. 700 km^2^. Elevation ranges from 375 to 3210 m and the vegetation has been and is still influenced by human activity with a mix of pastures and forests from the bottom of the valley up to the treeline at ca. 2100 m.

Our dataset contained 3988 vegetation plots with both forested areas^53^ (3076 plots; including clearings and very lose stands of trees at the treeline) and open grasslands^54^ (912 plots). The vegetation plots covered an area of 314 m^2^ (r = 10 m) in the forests and of 64 m^2^ (8 × 8 m) in the open grasslands. Although, the two subsets had different plot sizes and sampling strategies (grid based for forest, random stratified for grasslands), we pooled all available data together to cover a maximum of plant species and habitats across the study area (for details on sampling see Hartmann *et al*.^53^ and Randin *et al*.^54^). To ensure that this pooling did not affect our tests, all analyses were also conducted separately for the two datasets (see Appendix S1).

In total, 1096 plant species were recorded but only 397 (35%) had more than 50 presence records throughout all plots, and were selected for modelling (see Appendix S2 for species list). This study thus concerned mainly the “most frequent” species with a regional prevalence higher than 0.012 (1.2%).

### Environmental data

Based on 30 years (1961–1990) of meteorological data from national weather stations, fourteen climatic variables (Table S5) were calculated according to the method described in Zimmermann & Kienast^44^ and Zimmerman *et al*.^55^. A digital elevation model (DEM) was used to spatially interpolate the climatic data and to create topographic variables (Table S5). Additionally, we used a soil pH map based on Buri *et al*.^43^. All environmental data had a spatial resolution of 25 m, a resolution similar to the areas used to calculate the ecological indicator values (EIV; see below). This allowed a straightforward comparison of different sets of predictors composed from environmental variables and EIVs.

For our species distribution models (SDMs) we selected the following six environmental variables: annual mean temperature, annual sum of precipitation, annual sum of radiation, topographic position, slope and soil pH. These variables were selected because: (1) they are expected to be of high ecophysiological significance^6,9,56^ as they cover the main factors necessary for plant life^57^ (i.e., temperature, water, light, soil); (2) are not highly correlated (<0.5 except for annual temperature and annual sum of precipitation, Table S6); and (3) have already been used in numerous studies dealing with large numbers of different species and taxonomic groups^14,28,40,58,59^.

To analyze the correlation of the ecological indicator values and the bio-climatic predictors, we used an extended set of 17 widely available environmental variables (Table S5).

### Ecological indicator values

In this study we used the ecological indicator values (EIVs) first developed by Landolt^17^ and later extended by Landolt *et al*.^19^. Landolt’s EIVs are largely similar to the more renown ones proposed by Ellenberg *et al*.^18^, but specifically adapted to the flora and environmental conditions of Switzerland. There are a large number of EIVs (for details see Landolt *et al*.^19^), but in this study we focused on six of the most commonly used, namely: **T** for temperature;, **M** for soil moisture/water availability;, **L** for light;, **K** for continentality;, **R** for soil pH; and **N** for soil nutrients.

Based on the species list of each vegetation plot, we calculated the mean Landolt EIV per plot/site (site EIV) according to Landolt *et al*.^19^. We decided to calculate the site EIV as averaged EIV across all species present at a given site not weighted by abundance (see Diekmann^25^ for a discussion of weighted versus unweighted averaged values) because (1) abundance data was not available for all sites and (2) our models (SDMs) only use presence/absence information. To prevent any circularity in the conclusion, the focal species (being modelled) was always excluded in the calculation of site EIV, resulting in slightly different predictor values for each species and plot^24^.

### Testing the explanatory power of indicator values in SDMs

We used the set of our six classical environmental variables (annual mean temperature, annual sum of precipitation, annual sum of radiation, topographic position, slope and soil pH) as a “reference model” (M_ref; Table 1). To test the alternative predictive power of site EIVs, we used a framework with  eight different sets of predictors for our SDMs (Fig. 1), replacing classical environmental variables by site EIVs (M_T, M_Soil, M_R, M_N, M_F, M_K, M_L, M_EIV; Table 1). Additionally, based on our correlation analysis we choose the set of the six bio-climatic predictors most strongly correlated with the six ecological indicator values (M_cor; Table 1). This set of predictors should minimize the difference between environmental variables and site EIV-based predictions by maximizing cross-correlations and should therefore represent a potentially optimal set of bio-climatic predictors.

All SDMs were run with the R package biomod2^60,61^ in R 3.1.2^62^ using generalized linear models (GLM), generalized additive models (GAM), surface range envelope (SRE), random forest (RF) and maximum entropy (MAXENT^63^) as modelling technique. We selected these techniques as they are the most commonly used in the literature and therefore allow representative results. We decided not to use an ensemble modelling approach^64^ as we were mostly interested in potential differences among model inputs rather than in obtaining the most accurate predictions.

To evaluate the models, we split the data into 70% as calibration data and 30% as evaluation data, the latter subset being never used in any step of the model building, therefore allowing independent “external” evaluation. Additionally, the calibration data set was split again randomly 10 times into 70%/30% to add internal evaluation (by repeated cross-validation). In both internal and external evaluation, we calculated the area under the curve^65^ (AUC), true skill statistic^66^ (TSS), Cohens kappa^67^ (KAPPA), the proportion of correct predictions (Accuracy) and (for GLM and GAM models only) the explained deviance^6^ (D^2^). We also compared the variable importance, estimated by permutation tests^60^, among all the different sets of predictors using a pairwise-wilcoxon-test with a holm correction for p-values. All the evaluation metrics presented in this study were calculated on the 30% of independent “external” evaluation data.

### Testing whether EIV could not simply be more complex combinations of the environmental variables

Additional to the field recorded site EIVs, we also used several techniques (GLM, GAM, GBM and RF) to model site EIVs in space based on the same bio-climatic predictors used for the SDMs (Table S5). This allowed us to assess if site EIVs can be modelled from the same environmental variables used in SDMs (using perhaps some more complex relationships), and thus do not bring much additional explanatory power than these environmental variables themselves, or if they provide new information that cannot be accounted for, even by more complex combinations of the environmental variables. To assess the model accuracy of the predicted site EIVs, we calculated the Pearson correlation and root square mean error of the predicted and field recorded site EIVs based on a 25-fold cross-validation (70/30% split).

## Supplementary information

Supplementary Information

## Data Availability

All data (species occurrences and predictors) are available on Dryad provisory doi:10.5061/dryad.qg4mj32^68^.

## References

[1] Franklin, J. *Mapping species distribution: spatial inference and prediction*. (Cambridge University Press, 2010).

[2] Peterson, A. T. *et al*. *Ecological Niches and Geographic Distributions*. (Princeton University Press, 2011).

[3] Guisan, A., Thuiller, W. & Zimmermann, N. E. *Habitat suitability and distribution models*. (Cambridge University Press, 2017).

[4] Austin, M. P. Searching for a model for use in vegetation analysis. *Vegetatio* **42**, 11–21, 10.1007/Bf00048865.

[5] James, F. C., Johnston, R. F., Wamer, N. O., Niemi, G. J. & Boecklen, W. J. The Grinnellian niche of the wood thrush. *Am*. *Nat*., 17–47.

[6] Guisan, A. & Zimmermann, N. E. Predictive habitat distribution models in ecology. *Ecol*. *Modell*. **135**, 147–186.

[7] Guisan, A. *et al*. Predicting species distributions for conservation decisions. *Ecol*. *Lett*. **16**, 1424–1435, 10.1111/Ele.12189. 10.1111/ele.12189 PMC4280402 24134332

[8] Araujo, M. B. & Guisan, A. Five (or so) challenges for species distribution modelling. *J*. *Biogeogr*. **33**, 1677–1688, 10.1111/j.1365-2699.2006.01584.x.

[9] Austin, M. P. & Van Niel, K. P. Improving species distribution models for climate change studies: variable selection and scale. *J*. *Biogeogr*. **38**, 1–8, 10.1111/j.1365-2699.2010.02416.x.

[10] Wilson, A. M. & Jetz, W. Remotely sensed high-resolution global cloud dynamics for predicting ecosystem and biodiversity distributions. *PLoS Biol*. **14**, e1002415. 10.1371/journal.pbio.1002415 PMC4816575 27031693

[11] Pekel, J.-F., Cottam, A., Gorelick, N. & Belward, A. S. High-resolution mapping of global surface water and its long-term changes. *Natur*e **54**0, 418–422. 10.1038/nature20584 27926733

[12] Pradervand, J. N., Dubuis, A., Pellissier, L., Guisan, A. & Randin, C. Very high resolution environmental predictors in species distribution models: Moving beyond topography? *Prog*. *Phys*. *Geog*. **38**, 79–96, 10.1177/0309133313512667.

[13] Lenoir, J., Hattab, T. & Pierre, G. Climatic microrefugia under anthropogenicclimate change: implications for species redistribution. *Ecography* **40**, 253–266.

[14] Mod, H. K., Scherrer, D., Luoto, M. & Guisan, A. What we use is not what we know: environmental predictors in plant distribution models. *J*. *Veg*. *Sci*. **27**, 1308–1322, 10.1111/jvs.12444.

[15] Soberón, J. & Arroyo-Peña, B. Are fundamental niches larger than the realized? Testing a 50-year-old prediction by Hutchinson. *Plos One* **12**, e0175138. 10.1371/journal.pone.0175138 PMC5389801 28403170

[16] Kearney, M. & Porter, W. P. Mapping the fundamental niche: Physiology, climate, and the distribution of a nocturnal lizard. *Ecology* **85**, 3119–3131, 10.1890/03-0820.

[17] Landolt, E. Okologische zeigerwerte zur Schweizer flora. *Veröff Geobot Inst ETH Stift Rübel* **64**.

[18] Ellenberg, H. Indicator values of vascular plants in centralEurope. *Scripta geobotanic*a **9**.

[19] Landolt, E. *et al*. *Flora indicativa: Ecological indicator values and Biological attributes of flora of Switzerland and the Alps*. 378 (Haupt Verlag, 2010).

[20] Moeslund, J. E. *et al*. Topographically controlled soil moisture drives plant diversity patterns within grasslands. *Biodivers*. *Conserv*. **22**, 2151–2166, 10.1007/s10531-013-0442-3.

[21] Hill, M. & Carey, P. Prediction of yield in the Rothamsted Park Grass Experiment by Ellenberg indicator values. *J*. *Veg*. *Sci*. **8**, 579–586.

[22] Tölgyesi, C., Bátori, Z. & Erdős, L. Using statistical tests on relative ecological indicator values to compare vegetation units–Different approaches and weighting methods. *Ecol*. *Indic*. **36**, 441–446.

[23] Ischer, M., Dubuis, A., Keller, R. & Vittoz, P. A better understanding of ecological conditions for Leontopodium alpinum Cassini in the Swiss Alps. *Folia Geobot*. **49**, 541–558, 10.1007/s12224-014-9190-8.

[24] Nieto-Lugilde, D. *et al*. Tree cover at fine and coarse spatial grains interacts with shade tolerance to shape plant species distributions across the Alps *Ecography* **37**, 1–12. 10.1111/ecog.00954 PMC4538783 26290621

[25] Diekmann, M. Species indicator values as an important tool in applied plant ecology–a review. *Basic Appl*. *Ecol*. **4**, 493–506.

[26] Wamelink, G., Joosten, V., Dobben, H. V. & Berendse, F. Validity of Ellenberg indicator values judged from physico‐chemical field measurements. *J*. *Veg*. *Sci*. **13**, 269–278.

[27] Cheddadi, R. *et al*. Temperature range shifts for three European tree species over the last 10,000 years. *Front*. *Plant*. *Sci*. **7**. 10.3389/fpls.2016.01581 PMC5078669 27826308

[28] Scherrer, D., Massy, S., Meier, S., Vittoz, P. & Guisan, A. Assessing and predicting shifts in mountain forest composition across 25 years of climate change. *Divers*. *Distrib*. **23**, 517–528.

[29] Lenoir, J., Gegout, J. C., Dupouey, J. L., Bert, D. & Svenning, J. C. Forest plant community changes during1989–2007 in response to climate warming in the Jura Mountains (France and Switzerland). *J*. *Veg*. *Sci*. **21**, 949–964, 10.1111/j.1654-1103.2010.01201.x.

[30] Oostermeijer, J. G. B. & van Swaay, C. A. M. The relationship between butterflies and environmental indicator values: a tool for conservation in a changing landscape. *Biol*. *Conserv*. **86**, 271–280.

[31] Coudun, C., Gegout, J. C., Piedallu, C. & Rameau, J. C. Soil nutritional factors improve models of plant species distribution: an illustration with Acer campestre (L.) in France. *J*. *Biogeogr*. **33**, 1750–1763.

[32] Lenoir, J. *et al*. Local temperatures inferred from plant communities suggest strong spatial buffering of climate warming across Northern Europe. *Glob*. *Chang*. *Biol*. **19**, 1470–1481, 10.1111/Gcb.12129. 10.1111/gcb.12129 23504984

[33] Austin, M. Species distribution models and ecological theory: A critical assessment and some possible new approaches. *Ecol*. *Modell*. **200**, 1–19, 10.1016/j.ecolmodel.2006.07.005.

[34] Scherrer, D. & Körner, C. Infra-red thermometry of alpine landscapes challenges climatic warming projections. *Glob*. *Chang*. *Biol*. **16**, 2602–2613, 10.1111/j.1365-2486.2009.02122.x.

[35] Scherrer, D. & Körner, C. Topographically controlled thermal-habitat differentiation buffers alpine plant diversity against climate warming. *J*. *Biogeogr*. **38**, 406–416, 10.1111/j.1365-2699.2010.02407.x.

[36] Niskanen, A., Luoto, M., Väre, H. & Heikkinen, R. K. Models of Arctic-alpine refugia highlight importance of climate and local topography. *Polar Biol*. 1–14.

[37] le Roux, P. C., Aalto, J. & Luoto, M. Soil moisture’s underestimated role in climate change impact modelling in low-energy systems. *Glob*. *Chang*. *Biol*. **19**, 2965–2975, 10.1111/gcb.12286. 10.1111/gcb.12286 23749628

[38] Kalan, P., Kosmelj, K., Taillie, C., Cedilnik, A. & Carson, J. H. Quantifying the efficiency of soil sampling designs: A multivariate approach. *Environ*. *Ecol*. *Stat*. **1**0, 469–482.

[39] Bertrand, R., Perez, V. & Gegout, J. C. Disregarding the edaphic dimension in species distribution models leads to the omission of crucial spatial information under climate change: the case of Quercus pubescens in France. *Glob*. *Chang*. *Biol*. **18**, 2648–2660, 10.1111/J.1365-2486.2012.02679.X.

[40] Dubuis, A. *et al*. Improving the prediction of plant species distribution and community composition by adding edaphic to topo-climatic variables. *J*. *Veg*. *Sci*. **24**, 593–606, 10.1111/jvs.12002.

[41] Mellert, K. H. *et al*. Soil water storage appears to compensate for climatic aridity at the xeric margin of European tree species distribution. *European Journal of Forest Research*, 1–14.

[42] Marage, D. & Gegout, J. C. Importance of soil nutrients in the distribution of forest communities on a large geographical scale. *Global Ecol*. *Biogeogr*. **18**, 88–97, 10.1111/j.1466-8238.2008.00428.x.

[43] Buri, A. *et al*. Soil factors improve predictions of plant species distribution in a mountain environment. *Prog*. *Phys*. *Geog*. **41**, 703–722.

[44] Zimmermann, N. E. & Kienast, F. Predictive mapping of alpine grasslands in Switzerland: Species versus community approach. *J*. *Veg*. *Sci*. **10**, 469–482.

[45] Moeslund, J. E. *et al*. Topographically controlled soil moisture is the primary driver of local vegetation patterns across a lowland region. *Ecosphere* **4**, 10.1890/Es13-00134.1.

[46] Lortie, C. J. *et al*. Rethinking plant community theory. *Oikos* **107**, 433–438.

[47] Guisan, A. & Rahbek, C. SESAM - a new framework integrating macroecological and species distribution models for predicting spatio-temporal patterns of species assemblages. *J*. *Biogeogr*. **38**, 1433–1444, 10.1111/j.1365-2699.2011.02550.x.

[48] D’Amen, M., Rahbek, C., Zimmermann, N. E. & Guisan, A. Spatial predictions at the community level: from current approaches to future frameworks. *Biol*. *Rev*. **92**, 169–187, 10.1111/brv.12222. 10.1111/brv.12222 26426308

[49] Fernandes, R. F., Scherrer, D. & Guisan, A. How much should one sample to accurately predict the distribution of species assemblages? A virtual community approach. *Ecol*. *Inform*. **48**, 125–134.

[50] Estes, L. D., Reillo, P. R., Mwangi, A. G., Okin, G. S. & Shugart, H. H. Remote sensing of structural complexity indices for habitat and species distribution modeling. *Remote Sens*. *Environ*. **114**, 792–804, 10.1016/j.rse.2009.11.016.

[51] Bradley, B. A. & Fleishman, E. Can remote sensing of land cover improve species distribution modelling? *J*. *Biogeogr*. **35**, 1158–1159, 10.1111/j.1365-2699.2008.01928.x.

[52] McGill, B. J. Matters of Scale. *Science* **328**, 575–576, 10.1126/science.1188528. 10.1126/science.1188528 20431001

[53] Hartmann, P., Fouvy, P. & Horisberger, D. L’Observatoire de l′écosystème forestier du canton de Vaud: espace de recherche appliquée| The Forest Ecosystem Observatory in Canton Vaud: a field of applied research. *Schweiz Z*. *Forst*. **160**, s2–s6.

[54] Randin, C. F. *et al*. Climate change and plant distribution: local models predict high-elevation persistence. *Glob*. *Chang*. *Biol*. **15**, 1557–1569, 10.1111/j.1365-2486.2008.01766.x.

[55] Zimmermann, N. E., Edwards, T. C., Moisen, G. G., Frescino, T. S. & Blackard, J. A. Remote sensing-based predictors improve distribution models of rare, early successional and broadleaf tree species in Utah. *J*. *Appl*. *Ecol*. **44**, 1057–1067, 10.1111/j.1365-2664.2007.01348.x. 10.1111/j.1365-2664.2007.01348.x PMC2368764 18642470

[56] Guisan, A. & Thuiller, W. Predicting species distribution: offering more than simple habitat models. *Ecol*. *Lett*. **8**, 993–1009, 10.1111/j.1461-0248.2005.00792.x. 10.1111/j.1461-0248.2005.00792.x 34517687

[57] Körner, C. *Alpine Plant Life: Functional Plant Ecology of High Mountain Ecosystem*. 2nd Edition edn, (Springer, 2003).

[58] D’Amen, M. *et al*. Using species richness and functional traits predictions to constrain assemblage predictions from stacked species distribution models. *J*. *Biogeogr*. **42**, 1255–1266, 10.1111/jbi.12485.

[59] Pottier, J. *et al*. The accuracy of plant assemblage prediction from species distribution models varies along environmental gradients. *Global Ecol*. *Biogeogr*. **22**, 52–63, 10.1111/j.1466-8238.2012.00790.x.

[60] Thuiller, W., Georges, D. & Engler, R. Biomod2: Ensemble platform for species distribution modeling. *R package version* **2**, r560.

[61] Thuiller, W., Lafourcade, B., Engler, R. & Araujo, M. B. BIOMOD - a platform for ensemble forecasting of species distributions. *Ecography* **32**, 369–373, 10.1111/j.1600-0587.2008.05742.x.

[62] R: A Language and Environment for Statistical Computing (R Foundation for Statistical Computing, Vienna, Austria, 2017).

[63] Phillips, S. J., Anderson, R. P. & Schapire, R. E. Maximum entropy modeling of species geographic distributions. *Ecol*. *Modell*. **190**, 231–259, 10.1016/j.ecolmodel.2005.03.026.

[64] Araujo, M. B. & New, M. Ensemble forecasting of species distributions. *Trends Ecol*. *Evol*. **22**, 42–47, 10.1016/j.tree.2006.09.010. 10.1016/j.tree.2006.09.010 17011070

[65] Fielding, A. H. & Bell, J. F. A review of methods for the assessment of prediction errors in conservation presence/absence models. *Environ*. *Conserv*. **24**, 38–49.

[66] Allouche, O., Tsoar, A. & Kadmon, R. Assessing the accuracy of species distribution models: prevalence, kappa and the true skill statistic (TSS). *J*. *Appl*. *Ecol*. **43**, 1223–1232, 10.1111/j.1365-2664.2006.01214.x.

[67] Cohen, J. A Coefficient of Agreement for Nominal Scales. *Educ*. *Psychol*. *Meas*. **20**, 37–46.

[68] Guisan, A. *et al*. Data from: Ecological indicator values reveal missing environmental predictors of species distributions. *Dryad Digital Repository*, 10.5061/dryad.qg4mj32 (2018).

