# Supplementary material for: Ecological indicator values reveal missing predictors of species distributions
Source: Sci Rep. 2019 Feb 28;9:3061. doi: 10.1038/s41598-019-39133-1 (PMC6395803; doi:10.1038/s41598-019-39133-1)
Supplement: Supplementary file 1 — Supplementary Information [file 41598_2019_39133_MOESM1_ESM.docx]

**Ecological indicator values reveal missing predictors of species distributions**

Daniel Scherrer* & Antoine Guisan

Scherrer, D. (corresponding author, daniel.scherrer@unil.ch)^a^

Guisan, A. (antoine.guisan@unil.ch)^a,b^

^a^ Department of Ecology and Evolution, University of Lausanne, Biophore, CH-1015 Lausanne, Switzerland
^b^ Institute of Earth Sciences, University of Lausanne, Géopolis, CH-1015 Lausanne, Switzerland

**Figure S1**

**
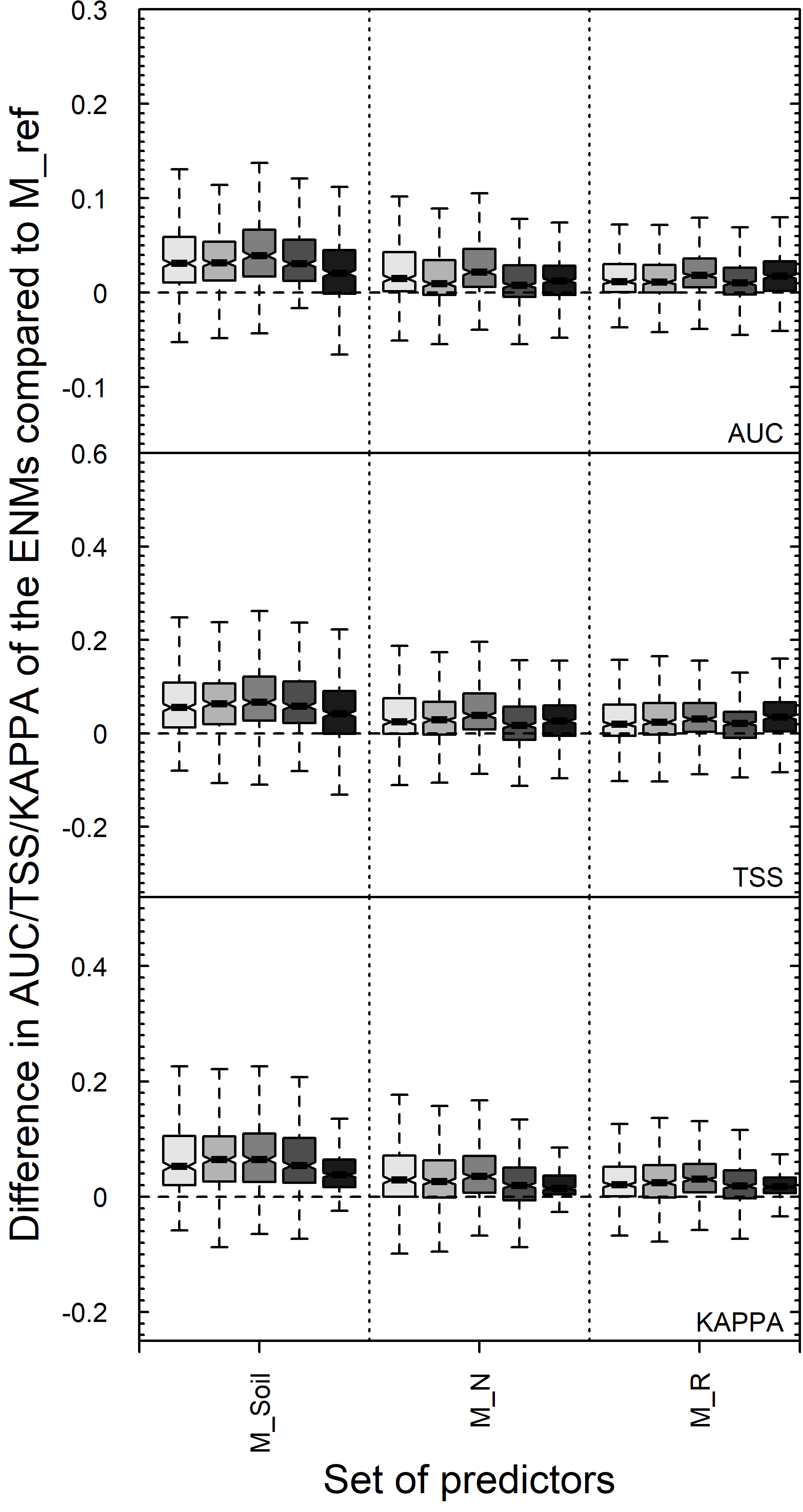
**

Fig. S1 Differences in AUC, TSS and KAPPA of the different set of soil predictors (M_Soil, M_R, M_N) compared to our reference model (M_ref, see Table 1 for details). The data shown is for all 397 species evaluated on an independent ‘external’ data set of 1193 vegetation plots. The different shades of grey from light to dark represent GLM, GAM, RF, MAXENT and SRE models. The boxes represent the median and the 25/75 percentile and the whiskers are 2 SD.

**Table S1**. Evaluation metrics of the different modelling techniques for the SDM predictor set M_ref (slope, topographic position, annual average temperature, annual sum of rainfall, annual sum of radiation, pH). All metrics are based on 397 plant species evaluated on an independent dataset of 1193 plots.

| Modelling technique | AUC  (mean ± sd) | TSS  (mean ± sd) | KAPPA  (mean ± sd) | Accuracy  (mean ± sd) |
| --- | --- | --- | --- | --- |
| GLM | 0.81 ± 0.09 | 0.49 ± 0.18 | 0.23 ± 0.14 | 0.93 ± 0.07 |
| GAM | 0.83 ± 0.08 | 0.50 ± 0.17 | 0.25 ± 0.14 | 0.93 ± 0.07 |
| RF | 0.80 ± 0.09 | 0.45 ± 0.19 | 0.23 ± 0.15 | 0.93 ± 0.07 |
| MaxEnt | 0.83 ± 0.09 | 0.51 ± 0.18 | 0.25 ± 0.14 | 0.93 ± 0.07 |
| SRE | 0.67 ± 0.08 | 0.34 ± 0.16 | 0.11 ± 0.10 | 0.93 ± 0.08 |

**Table S2.** Evaluation statistics of the different modelling techniques for all used SDM predictor sets. All evaluations are based on an independent dataset of 1193 plots.

| Predictor set | Modelling technique | AUC  (mean ± sd) | TSS  (mean ± sd) | KAPPA  (mean ± sd) | Accuracy  (mean ± sd) |
| --- | --- | --- | --- | --- | --- |
| M_ref | GLM | 0.81 ± 0.09 | 0.49 ± 0.18 | 0.23 ± 0.14 | 0.93 ± 0.07 |
|  | GAM | 0.83 ± 0.08 | 0.50 ± 0.17 | 0.25 ± 0.14 | 0.93 ± 0.07 |
|  | RF | 0.80 ± 0.09 | 0.45 ± 0.19 | 0.23 ± 0.15 | 0.93 ± 0.07 |
|  | MaxEnt | 0.83 ± 0.09 | 0.51 ± 0.18 | 0.25 ± 0.14 | 0.93 ± 0.07 |
|  | SRE | 0.67 ± 0.08 | 0.34 ± 0.16 | 0.11 ± 0.10 | 0.93 ± 0.08 |
| M_cor | GLM | 0.82 ± 0.09 | 0.50 ± 0.17 | 0.24 ± 0.14 | 0.93 ± 0.07 |
|  | GAM | 0.83 ± 0.08 | 0.50 ± 0.16 | 0.25 ± 0.14 | 0.93 ± 0.07 |
|  | RF | 0.80 ± 0.09 | 0.47 ± 0.18 | 0.24 ± 0.14 | 0.93 ± 0.07 |
|  | MaxEnt | 0.83 ± 0.09 | 0.52 ± 0.18 | 0.25 ± 0.15 | 0.93 ± 0.07 |
|  | SRE | 0.68 ± 0.08 | 0.36 ± 0.17 | 0.12 ± 0.11 | 0.93 ± 0.07 |
| M_Soil | GLM | 0.85 ± 0.08 | 0.56 ± 0.16 | 0.30 ± 0.15 | 0.94 ± 0.06 |
|  | GAM | 0.86 ± 0.07 | 0.57 ± 0.16 | 0.32 ± 0.15 | 0.94 ± 0.06 |
|  | RF | 0.85 ± 0.08 | 0.53 ± 0.16 | 0.31 ± 0.14 | 0.94 ± 0.06 |
|  | MaxEnt | 0.87 ± 0.07 | 0.58 ± 0.16 | 0.31 ± 0.14 | 0.94 ± 0.06 |
|  | SRE | 0.70 ± 0.07 | 0.39 ± 0.13 | 0.16 ± 0.11 | 0.93 ± 0.07 |
| M_N | GLM | 0.84 ± 0.08 | 0.53 ± 0.17 | 0.27 ± 0.14 | 0.94 ± 0.06 |
|  | GAM | 0.84 ± 0.08 | 0.53 ± 0.16 | 0.28 ± 0.14 | 0.93 ± 0.07 |
|  | RF | 0.83 ± 0.08 | 0.50 ± 0.17 | 0.28 ± 0.14 | 0.93 ± 0.06 |
|  | MaxEnt | 0.84 ± 0.08 | 0.53 ± 0.15 | 0.27 ± 0.13 | 0.93 ± 0.07 |
|  | SRE | 0.69 ± 0.07 | 0.37 ± 0.15 | 0.13 ±0.10 | 0.93 ± 0.07 |
| M_R | GLM | 0.83 ± 0.09 | 0.52 ± 0.17 | 0.26 ± 0.14 | 0.93 ± 0.07 |
|  | GAM | 0.84 ± 0.08 | 0.53 ± 0.16 | 0.27 ± 0.14 | 0.93 ± 0.06 |
|  | RF | 0.82 ± 0.08 | 0.49 ± 0.17 | 0.27 ± 0.15 | 0.94 ± 0.06 |
|  | MaxEnt | 0.84 ± 0.08 | 0.53 ± 0.16 | 0.27 + 0.13 | 0.93 ± 0.07 |
|  | SRE | 0.69 ± 0.07 | 0.37 ± 0.15 | 0.13 ± 0.10 | 0.93 ± 0.08 |
| M_M | GLM | 0.85 ± 0.09 | 0.55 ± 0.17 | 0.28 ± 0.14 | 0.94 ± 0.06 |
|  | GAM | 0.86 ± 0.08 | 0.55 ± 0.16 | 0.29 ± 0.14 | 0.94 ± 0.06 |
|  | RF | 0.83 ± 0.08 | 0.51 ± 0.17 | 0.28 ± 0.15 | 0.94 ± 0.06 |
|  | MaxEnt | 0.86 ± 0.08 | 0.57 ± 0.17 | 0.30 ± 0.14 | 0.94 ± 0.06 |
|  | SRE | 0.68 ± 0.07 | 0.37 ± 0.15 | 0.13 ± 0.10 | 0.93 ± 0.07 |
| M_K | GLM | 0.85 ± 0.09 | 0.56 ± 0.17 | 0.27 ± 0.14 | 0.93 ± 0.07 |
|  | GAM | 0.86 ± 0.08 | 0.57 ± 0.16 | 0.29 ± 0.14 | 0.93 ± 0.06 |
|  | RF | 0.83 ± 0.09 | 0.52 ± 0.17 | 0.27 ± 0.14 | 0.94 ± 0.06 |
|  | MaxEnt | 0.87 ± 0.08 | 0.58 ± 0.17 | 0.29 ± 0.14 | 0.94 ± 0.06 |
|  | SRE | 0.72 ± 0.07 | 0.44 ± 0.15 | 0.15 ± 0.10 | 0.93 ± 0.08 |
| M_L | GLM | 0.87 ± 0.09 | 0.60 ± 0.18 | 0.32 ± 0.16 | 0.94 ± 0.06 |
|  | GAM | 0.88 ± 0.08 | 0.61 ± 0.17 | 0.34 ± 0.16 | 0.94 ± 0.06 |
|  | RF | 0.86 ± 0.08 | 0.58 ± 0.18 | 0.32 ± 0.16 | 0.94 ± 0.06 |
|  | MaxEnt | 0.88 ± 0.08 | 0.63 ± 0.17 | 0.34 ± 0.16 | 0.94 ± 0.06 |
|  | SRE | 0.73 ± 0.08 | 0.47 ± 0.16 | 0.21 ± 0.14 | 0.93 ± 0.07 |
| M_EIV | GLM | 0.90 ± 0.06 | 0.67 ± 0.15 | 0.39 ± 0.16 | 0.94 ± 0.06 |
|  | GAM | 0.91 ± 0.06 | 0.67 ± 0.15 | 0.40 ± 0.16 | 0.94 ± 0.06 |
|  | RF | 0.89 ± 0.07 | 0.63 ± 0.16 | 0.38 ± 0.16 | 0.94 ± 0.06 |
|  | MaxEnt | 0.91 ± 0.06 | 0.68 ± 0.15 | 0.40 ± 0.15 | 0.94 ± 0.06 |
|  | SRE | 0.76 ± 0.06 | 0.52 ± 0.13 | 0.28 ± 0.15 | 0.93 ± 0.07 |

**Table S3**. Pearson correlation of field based and predicted site’s EIV using different modeling techniques.

| Modeling  Technique | T | K | L | M | R | N |
| --- | --- | --- | --- | --- | --- | --- |
| GLM | 0.94 | 0.41 | 0.67 | 0.51 | 0.66 | 0.54 |
| GAM | 0.96 | 0.49 | 0.78 | 0.61 | 0.75 | 0.69 |
| GBM | 0.96 | 0.50 | 0.79 | 0.63 | 0.75 | 0.68 |
| RF | 0.96 | 0.53 | 0.79 | 0.64 | 0.76 | 0.68 |

**Table S4.** Pearson correlation (a) and root mean square error (b) of field based and predicted ecological indicator values

(a)

| Modeling  Technique | T | K | L | M | R | N |
| --- | --- | --- | --- | --- | --- | --- |
| GLM | 0.94 | 0.41 | 0.67 | 0.51 | 0.66 | 0.54 |
| GAM | 0.96 | 0.49 | 0.78 | 0.61 | 0.75 | 0.69 |
| GBM | 0.96 | 0.50 | 0.79 | 0.63 | 0.75 | 0.68 |
| RF | 0.96 | 0.53 | 0.79 | 0.64 | 0.76 | 0.68 |

(b)

| Modeling  Technique | T | K | L | M | R | N |
| --- | --- | --- | --- | --- | --- | --- |
| GLM | 0.17 | 0.22 | 0.52 | 0.22 | 0.30 | 0.29 |
| GAM | 0.13 | 0.21 | 0.44 | 0.20 | 0.27 | 0.26 |
| GBM | 0.14 | 0.21 | 0.43 | 0.20 | 0.26 | 0.26 |
| RF | 0.13 | 0.20 | 0.43 | 0.20 | 0.25 | 0.25 |

**Table S5**. Topographic, climatic and soil variables used in this study. Average values of a 30 year period (1961-1990).

| Variables | Units | Details | References |
| --- | --- | --- | --- |
| Cloudines | % | Monthly mean cloudiness | ^1^ |
| Continentality | unitless | Continentality indices | ^1^ |
| Degreedays | °C | Annual degreedays with 5 °C threshold | ^1^ |
| Evapotranspiration | mm/month | Monthly potential evapotranspiration | ^1^ |
| Precipitation days | Number of days | Number of precipitation days | ^1^ |
| Precipitation | Mm/month | Monthly mean precipitation sum | ^1^ |
| Diffuse radiation | KJ/day | Monthly potential diffuse shortwave radiation | ^1^ |
| Direct radiation | KJ/day | Monthly potential direct shortwave radiation | ^1^ |
| Total radiation | KJ/day | Monthly global potential shortwave radiation | ^1^ |
| Seasonal frost | Number of days | Annual average of frost days during growing season | ^1^ |
| Site water balance | mm/year | Annual average site water balance | ^1^ |
| Moisture index | mm/month | Monthly moisture index. P-ETP | ^1^ |
| Moisture balance | mm/month | Monthly moisture balance: P/ETP- 1 | ^1^ |
| Average temperature | °C | Monthly average temperature | ^1^ |
| Topographic position | Unitless | Topographic position | ^2^ |
| Slope | ° | Slope inclination | ^2^ |
| pH | Unitless | Modelled soil pH | (Aline et al?) |

**Table S6**. Correlation of the variables used in the SDMs. (a) Correlation of the predictors used in the GIS models. (b) Correlation of the predictors based on the Landolt indicator values.

**(a)**

|  | TaveY | PrecY | SradY | pH | Topo | Slope |
| --- | --- | --- | --- | --- | --- | --- |
| TaveY | 1 |  |  |  |  |  |
| PrecY | -0.92 | 1 |  |  |  |  |
| SradY | 0.07 | -0.07 | 1 |  |  |  |
| pH | 0.06 | -0.08 | -0.19 | 1 |  |  |
| Topo | -0.24 | 0.24 | 0.15 | -0.47 | 1 |  |
| Slope | -0.11 | 0.09 | -0.12 | 0.07 | 0.08 | 1 |

**(b)**

|  | T | M | L | R | K | N |
| --- | --- | --- | --- | --- | --- | --- |
| T | 1 |  |  |  |  |  |
| M | -0.20 | 1 |  |  |  |  |
| L | -0.63 | -0.28 | 1 |  |  |  |
| R | -0.09 | -0.34 | 0.39 | 1 |  |  |
| K | -0.15 | -0.69 | 0.63 | 0.34 | 1 |  |
| N | 0.51 | 0.49 | -0.47 | -0.36 | -0.45 | 1 |

**Appendix 1: Separate analysis for forest and grassland plots**


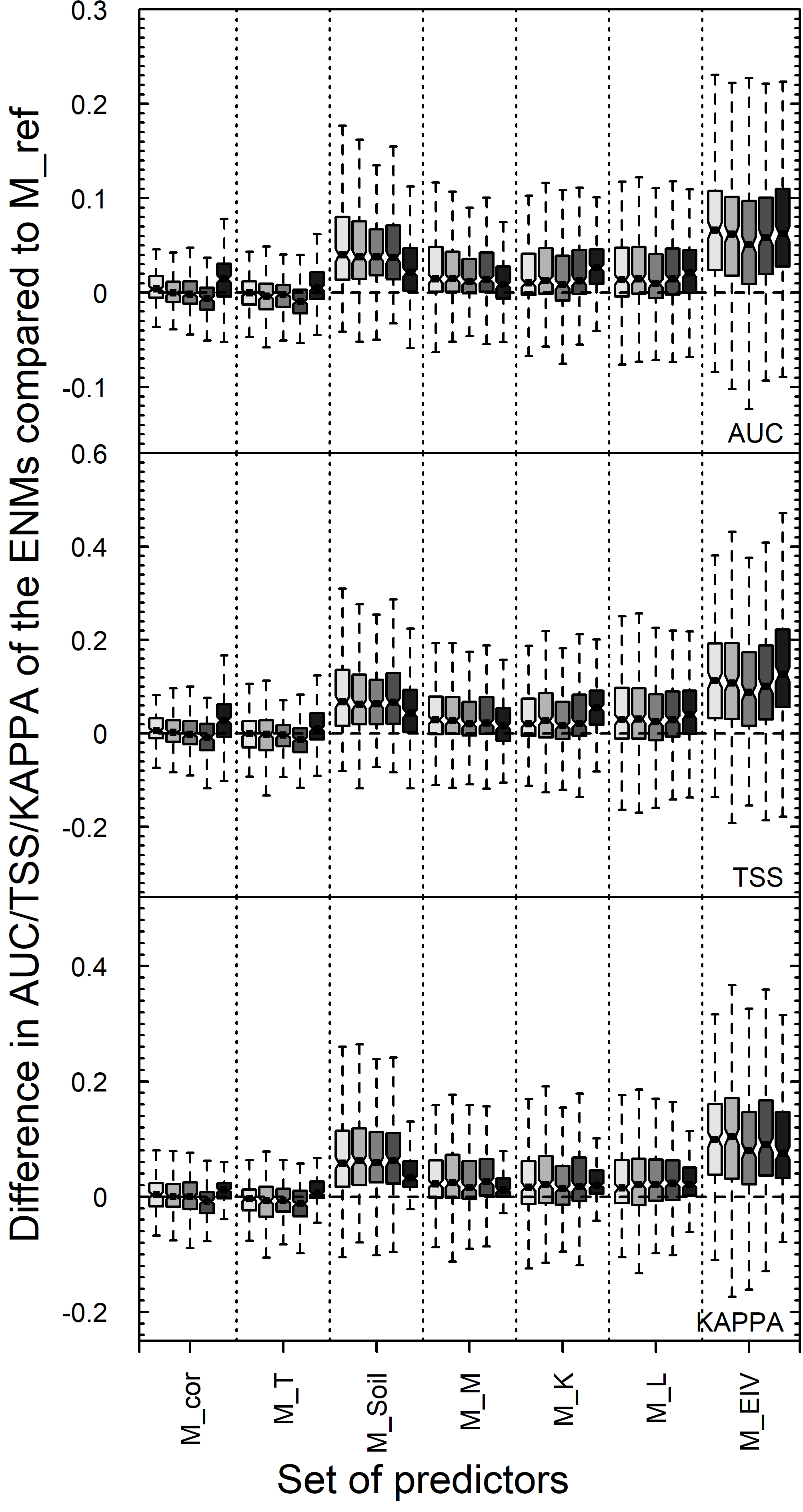


**Fig. A1S1.** Differences in AUC, TSS and KAPPA of the different set of predictors compared to our reference model (M_ref, see Table 1 for details). The data shown is for 260 **forest** species evaluated on an independent ‘external’ data set of 923 vegetation plots. The different shades of grey from light to dark represent GLM, GAM, RF, MAXENT and SRE models. The boxes represent the median and the 25/75 percentile and the whiskers are 2 SD.


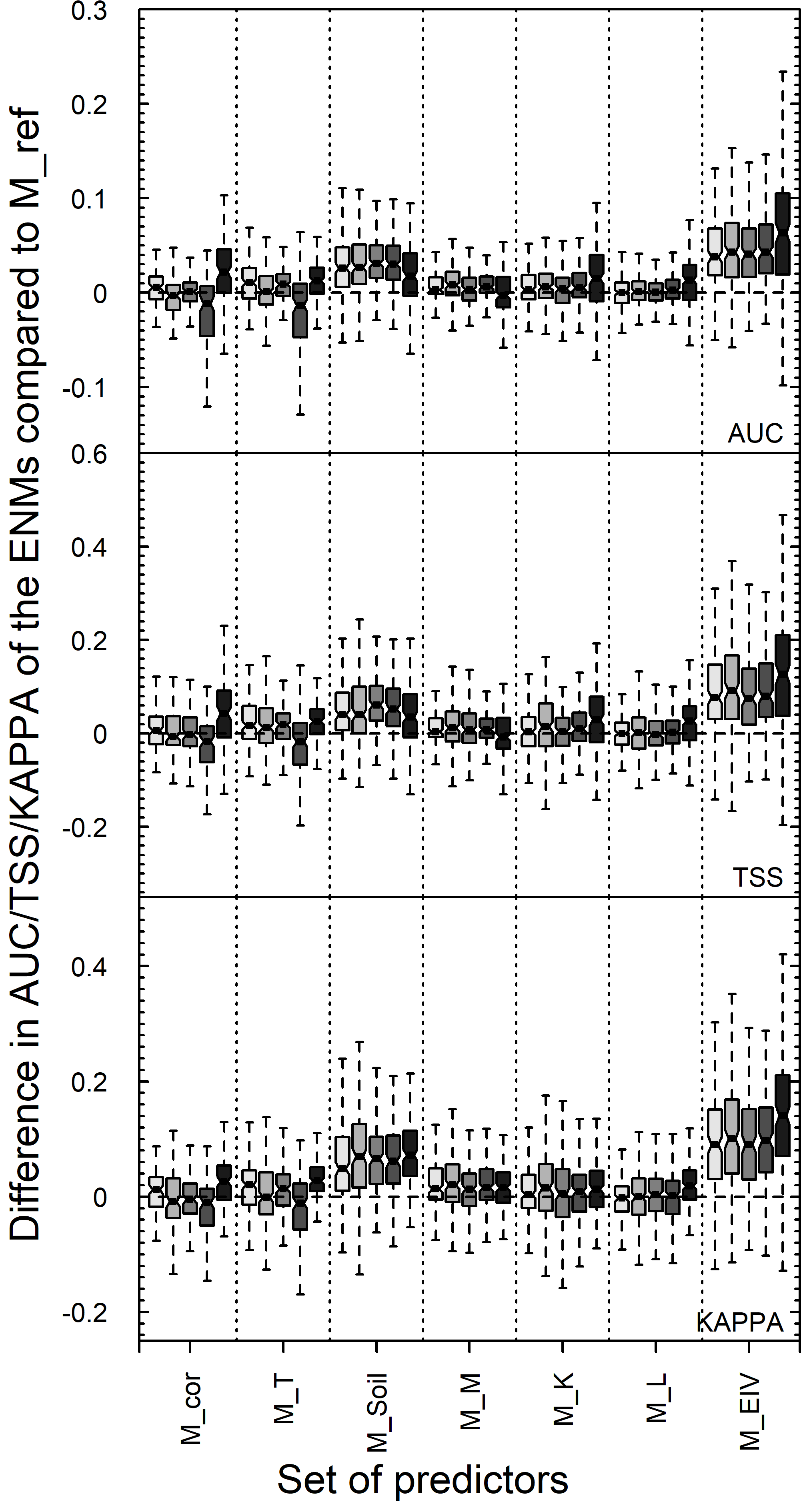


**Fig. A1S2**. Differences in AUC, TSS and KAPPA of the different set of predictors compared to our reference model (M_ref, see Table 1 for details). The data shown is for 178 **open grassland** species evaluated on an independent ‘external’ data set of 273 vegetation plots. The different shades of grey from light to dark represent GLM, GAM, RF, MAXENT and SRE models. The boxes represent the median and the 25/75 percentile and the whiskers are 2 SD.


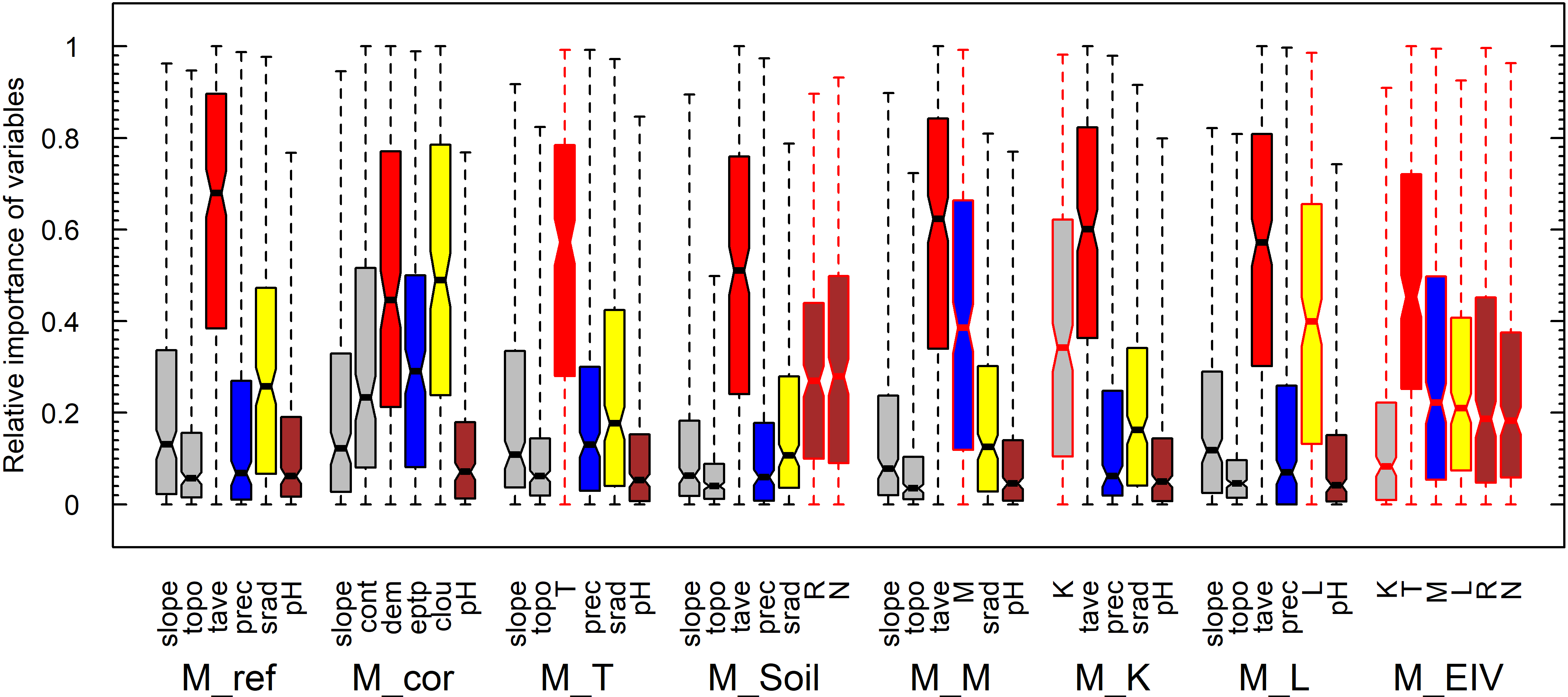


**Fig. A1S3**. The relative importance of variables of sets of predictors for the 260 **forest plot species** (see Table 1 for details). The different colors represent different categories of predictors: grey for topographic, red for temperature, blue for water, yellow for light and brown for soil. The bars outlined in black represent environmental predictors and the bars outlined in red represent site’s EIV. The boxes represent the median and the 25/75 percentile and the whiskers are 2 SD.


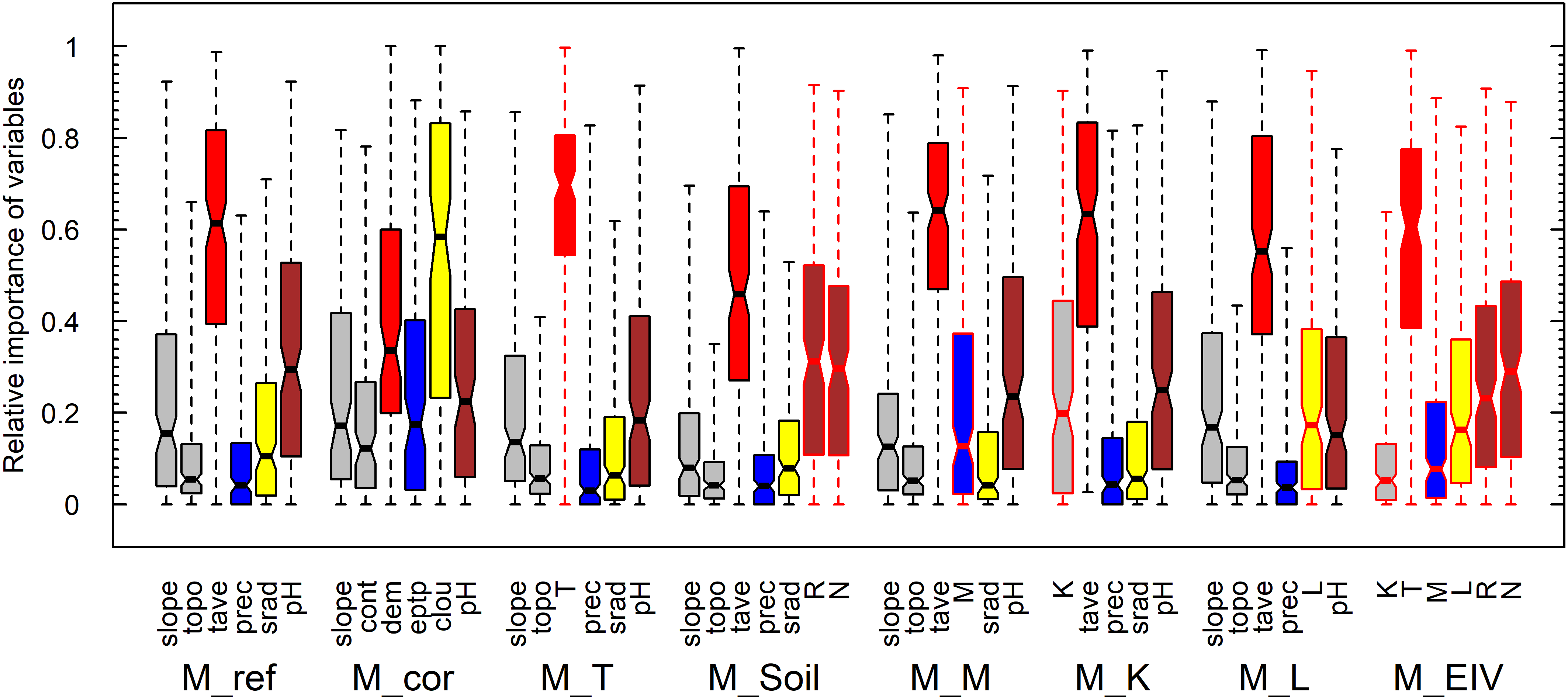


**Fig. A1S4**. The relative importance of variables of sets of predictors for the 178 **grassland plot species** (see Table 1 for details). The different colors represent different categories of predictors: grey for topographic, red for temperature, blue for water, yellow for light and brown for soil. The bars outlined in black represent environmental predictors and the bars outlined in red represent site’s EIV. The boxes represent the median and the 25/75 percentile and the whiskers are 2 SD.

**Appendix 2**: List of the 397 plant species used for the SDM models (more than 50 presences).

| *Abies alba* | *Euphrasia minima* | *Poa minor* |
| --- | --- | --- |
| *Acer campestre* | *Euphrasia rostkoviana* | *Poa nemoralis* |
| *Acer opalus* | *Euphrasia salisburgensis* | *Poa pratensis* |
| *Acer platanoides* | *Fagus sylvatica* | *Poa trivialis* |
| *Acer pseudoplatanus* | *Festuca altissima* | *Polygala alpestris* |
| *Achillea macrophylla* | *Festuca heterophylla* | *Polygala chamaebuxus* |
| *Achillea millefolium* | *Festuca quadriflora* | *Polygala vulgaris* |
| *Acinos alpinus* | *Festuca rubra* | *Polygonatum multiflorum* |
| *Aconitum lycoctonum aggr.* | *Festuca violacea* | *Polygonatum odoratum* |
| *Aconitum napellus aggr.* | *Filipendula ulmaria* | *Polygonatum verticillatum* |
| *Aconitum paniculatum* | *Fragaria vesca* | *Polygonum bistorta* |
| *Actaea spicata* | *Fraxinus excelsior* | *Polygonum viviparum* |
| *Adenostyles alliariae* | *Galeopsis tetrahit* | *Polypodium vulgare* |
| *Adenostyles glabra* | *Galium album* | *Polystichum aculeatum* |
| *Adoxa moschatellina* | *Galium anisophyllon* | *Polystichum lonchitis* |
| *Aegopodium podagraria* | *Galium aparine* | *Potentilla aurea* |
| *Agrostis capillaris* | *Galium megalospermum* | *Potentilla crantzii* |
| *Agrostis stolonifera* | *Galium mollugo* | *Potentilla erecta* |
| *Ajuga reptans* | *Galium odoratum* | *Potentilla sterilis* |
| *Allium ursinum* | *Galium pumilum* | *Prenanthes purpurea* |
| *Alnus incana* | *Galium rotundifolium* | *Primula acaulis* |
| *Alnus viridis* | *Gentiana asclepiadea* | *Primula auricula* |
| *Androsace chamaejasme* | *Gentiana campestris* | *Primula elatior* |
| *Anemone narcissiflora* | *Gentiana lutea* | *Pritzelago alpina* |
| *Anemone nemorosa* | *Gentiana purpurea* | *Prunella grandiflora* |
| *Angelica sylvestris* | *Gentiana verna* | *Prunella vulgaris* |
| *Anthericum ramosum* | *Geranium sylvaticum* | *Prunus avium* |
| *Anthoxanthum odoratum* | *Geum montanum* | *Pteridium aquilinum* |
| *Anthriscus sylvestris* | *Geum rivale* | *Pulmonaria officinalis* |
| *Anthyllis vulneraria aggr.* | *Geum urbanum* | *Pulsatilla alpina* |
| *Aposeris foetida* | *Glechoma hederacea* | *Quercus petraea* |
| *Aquilegia atrata* | *Globularia cordifolia* | *Quercus robur* |
| *Arabis alpina* | *Globularia nudicaulis* | *Ranunculus aconitifolius* |
| *Arabis hirsuta* | *Gymnadenia conopsea aggr.* | *Ranunculus alpestris* |
| *Arnica montana* | *Gymnocarpium dryopteris* | *Ranunculus bulbosus* |
| *Arrhenatherum elatius* | *Gymnocarpium robertianum* | *Ranunculus lanuginosus* |
| *Arum maculatum* | *Gypsophila repens* | *Ranunculus montanus* |
| *Aruncus dioicus* | *Hedera helix* | *Ranunculus nemorosus* |
| *Asplenium trichomanes* | *Hedysarum hedysaroides* | *Ranunculus platanifolius* |
| *Asplenium viride* | *Helictotrichon pubescens* | *Ranunculus repens* |
| *Aster bellidiastrum* | *Helictotrichon versicolor* | *Rhamnus alpina* |
| *Astrantia major* | *Helleborus foetidus* | *Rhinanthus alectorolophus* |
| *Astrantia minor* | *Hepatica nobilis* | *Rhododendron ferrugineum* |
| *Athyrium filix-femina* | *Heracleum sphondylium aggr.* | *Ribes alpinum* |
| *Bartsia alpina* | *Hieracium bifidum* | *Ribes petraeum* |
| *Bellis perennis* | *Hieracium lactucella* | *Rosa arvensis* |
| *Betula pendula* | *Hieracium prenanthoides* | *Rosa corymbifera* |
| *Blechnum spicant* | *Hieracium vogesiacum* | *Rosa pendulina* |
| *Brachypodium pinnatum* | *Hippocrepis comosa* | *Rubus caesius* |
| *Briza media* | *Hippocrepis emerus* | *Rubus idaeus* |
| *Bromus benekenii* | *Holcus lanatus* | *Rubus saxatilis* |
| *Bromus erectus* | *Homogyne alpina* | *Rumex acetosa* |
| *Calamagrostis varia* | *Hordelymus europaeus* | *Rumex alpestris* |
| *Caltha palustris* | *Hypericum maculatum* | *Rumex crispus* |
| *Campanula barbata* | *Hypericum montanum* | *Salix appendiculata* |
| *Campanula cochleariifolia* | *Hypochaeris radicata* | *Salix caprea* |
| *Campanula rhomboidalis* | *Ilex aquifolium* | *Salix retusa* |
| *Campanula rotundifolia* | *Impatiens noli-tangere* | *Salvia glutinosa* |
| *Campanula scheuchzeri* | *Juglans regia* | *Sambucus nigra* |
| *Campanula trachelium* | *Juncus effusus* | *Sambucus racemosa* |
| *Cardamine heptaphylla* | *Juniperus communis* | *Sanguisorba minor* |
| *Cardamine pentaphyllos* | *Juniperus nana* | *Sanicula europaea* |
| *Cardamine pratensis* | *Knautia arvensis* | *Saxifraga aizoides* |
| *Carduus defloratus* | *Knautia dipsacifolia* | *Saxifraga cuneifolia* |
| *Carex alba* | *Laburnum alpinum* | *Saxifraga oppositifolia* |
| *Carex caryophyllea* | *Lamium montanum* | *Saxifraga paniculata* |
| *Carex digitata* | *Larix decidua* | *Saxifraga rotundifolia* |
| *Carex ferruginea* | *Laserpitium latifolium* | *Scabiosa columbaria* |
| *Carex flacca* | *Laserpitium siler* | *Scabiosa lucida* |
| *Carex humilis* | *Lathyrus pratensis* | *Scrophularia nodosa* |
| *Carex montana* | *Lathyrus vernus* | *Sedum atratum* |
| *Carex ornithopoda* | *Leontodon autumnalis aggr.* | *Selaginella selaginoides* |
| *Carex pallescens* | *Leontodon helveticus* | *Senecio doronicum* |
| *Carex sempervirens* | *Leucanthemum vulgare aggr.* | *Senecio ovatus* |
| *Carex sylvatica* | *Ligusticum mutellina* | *Sesleria caerulea* |
| *Carlina simplex* | *Ligustrum vulgare* | *Silene acaulis* |
| *Carpinus betulus* | *Lilium martagon* | *Silene dioica* |
| *Carum carvi* | *Linum catharticum* | *Soldanella alpina* |
| *Castanea sativa* | *Listera ovata* | *Sorbus aria* |
| *Centaurea jacea* | *Lolium perenne* | *Sorbus aucuparia* |
| *Centaurea montana* | *Lonicera alpigena* | *Sorbus chamaemespilus* |
| *Cephalanthera damasonium* | *Lonicera caerulea* | *Sorbus mougeotii* |
| *Cephalanthera longifolia* | *Lonicera nigra* | *Stachys alpina* |
| *Cerastium latifolium* | *Lonicera xylosteum* | *Stachys officinalis* |
| *Cerastium vulgare* | *Lotus corniculatus* | *Stachys sylvatica* |
| *Cicerbita alpina* | *Luzula campestris* | *Stellaria graminea* |
| *Circaea lutetiana* | *Luzula luzulina* | *Stellaria nemorum* |
| *Cirsium acaule* | *Luzula multiflora* | *Tamus communis* |
| *Cirsium oleraceum* | *Luzula nivea* | *Taraxacum officinale* |
| *Cirsium palustre* | *Luzula pilosa* | *Taxus baccata* |
| *Cirsium spinosissimum* | *Luzula sylvatica* | *Thalictrum aquilegiifolium* |
| *Clematis vitalba* | *Lysimachia nemorum* | *Thesium alpinum* |
| *Clinopodium vulgare* | *Maianthemum bifolium* | *Thlaspi rotundifolium* |
| *Colchicum autumnale* | *Medicago lupulina* | *Thymus polytrichus* |
| *Convallaria majalis* | *Melampyrum sylvaticum* | *Thymus pulegioides* |
| *Cornus mas* | *Melica nutans* | *Tilia cordata* |
| *Cornus sanguinea* | *Melica uniflora* | *Tilia platyphyllos* |
| *Coronilla vaginalis* | *Melittis melissophyllum* | *Tofieldia calyculata* |
| *Corylus avellana* | *Mercurialis perennis* | *Tragopogon orientalis* |
| *Cotoneaster integerrimus* | *Milium effusum* | *Trifolium badium* |
| *Cotoneaster tomentosus* | *Moehringia muscosa* | *Trifolium medium* |
| *Crepis aurea* | *Mycelis muralis* | *Trifolium pratense* |
| *Crepis biennis* | *Myosotis alpestris* | *Trifolium repens* |
| *Crepis paludosa* | *Myosotis scorpioides* | *Trifolium thalii* |
| *Crepis pyrenaica* | *Myosotis sylvatica* | *Trisetum flavescens* |
| *Crocus albiflorus* | *Nardus stricta* | *Trollius europaeus* |
| *Cruciata laevipes* | *Neottia nidus-avis* | *Tussilago farfara* |
| *Cynosurus cristatus* | *Oreopteris limbosperma* | *Ulmus glabra* |
| *Cystopteris fragilis* | *Origanum vulgare* | *Urtica dioica aggr* |
| *Dactylis glomerata* | *Orthilia secunda* | *Vaccinium gaultherioides* |
| *Dactylorhiza maculata* | *Oxalis acetosella* | *Vaccinium myrtillus* |
| *Daphne laureola* | *Paris quadrifolia* | *Vaccinium vitis idaea aggr.* |
| *Daphne mezereum* | *Parnassia palustris* | *Valeriana dioica* |
| *Daucus carota* | *Pedicularis foliosa* | *Valeriana montana* |
| *Deschampsia cespitosa* | *Pedicularis verticillata* | *Valeriana tripteris* |
| *Digitalis lutea* | *Petasites albus* | *Veratrum lobelianum* |
| *Dryas octopetala* | *Petasites hybridus* | *Veronica alpina* |
| *Dryopteris carthusiana* | *Peucedanum ostruthium* | *Veronica aphylla* |
| *Dryopteris dilatata* | *Phleum hirsutum* | *Veronica arvensis* |
| *Dryopteris filix-mas* | *Phleum pratense* | *Veronica chamaedrys* |
| *Dryopteris oreades* | *Phleum rhaeticum* | *Veronica officinalis* |
| *Elymus caninus* | *Phyteuma orbiculare* | *Veronica urticifolia* |
| *Epilobium alpestre* | *Phyteuma spicatum* | *Viburnum lantana* |
| *Epilobium angustifolium* | *Picea abies* | *Viburnum opulus* |
| *Epilobium montanum* | *Pimpinella major aggr.* | *Vicia cracca* |
| *Epipactis atrorubens* | *Pinus sylvestris aggr.* | *Vicia sepium aggr* |
| *Epipactis helleborine* | *Plantago alpina* | *Vicia sylvatica* |
| *Equisetum arvense* | *Plantago atrata* | *Vincetoxicum hirundinaria* |
| *Equisetum sylvaticum* | *Plantago lanceolata* | *Viola biflora* |
| *Euonymus europaeus* | *Plantago major* | *Viola hirta* |
| *Euphorbia amygdaloides* | *Plantago media* | *Viola reichenbachiana* |
| *Euphorbia cyparissias* | *Platanthera bifolia* |  |
| *Euphorbia dulcis* | *Poa alpina* |  |

**References**

1 Zimmermann, N. E. & Kienast, F. Predictive mapping of alpine grasslands in Switzerland: Species versus community approach. *J. Veg. Sci.* **10**, 469-482

2 Zimmermann, N. E., Edwards, T. C., Moisen, G. G., Frescino, T. S. & Blackard, J. A. Remote sensing-based predictors improve distribution models of rare, early successional and broadleaf tree species in Utah. *J. Appl. Ecol.* **44**, 1057-1067, doi:10.1111/j.1365-2664.2007.01348.x
